# Maternal Supplementation with Small-Quantity Lipid-Based Nutrient Supplements Compared with Multiple Micronutrients, but Not with Iron and Folic Acid, Reduces the Prevalence of Low Gestational Weight Gain in Semi-Urban Ghana: A Randomized Controlled Trial^1^,^2^,^3^

**DOI:** 10.3945/jn.116.242909

**Published:** 2017-03-08

**Authors:** Seth Adu-Afarwuah, Anna Lartey, Harriet Okronipa, Per Ashorn, Ulla Ashorn, Mamane Zeilani, Mary Arimond, Stephen A Vosti, Kathryn G Dewey

**Affiliations:** 4 Department of Nutrition and Food Science, University of Ghana, Legon, Accra, Ghana;; 5 Center for Child Health Research, University of Tampere School of Medicine and Tampere University Hospital, Tampere, Finland;; 6 Nutriset S.A.S., Malaunay, France;; 7 Program in International and Community Nutrition, Department of Nutrition, and; 8 Department of Agricultural and Resource Economics, University of California, Davis, Davis, CA

**Keywords:** gestational weight gain, lipid-based nutrient supplements, maternal supplementation, multiple micronutrient supplementation, iron and folic acid supplementation

## Abstract

**Background:** It is unclear whether maternal supplementation with small-quantity lipid-based nutrient supplements (SQ-LNSs; 118 kcal/d) affects maternal weight.

**Objective:** We compared several secondary anthropometric measures between 3 groups of women in the iLiNS (International Lipid-based Nutrient Supplements)-DYAD trial in Ghana.

**Methods:** Women (*n* = 1320; <20 wk of gestation) were randomly assigned to receive 60 mg Fe + 400 μg folic acid/d (IFA), 18 vitamins and minerals/d [multiple micronutrients (MMNs)], or 20 g SQ-LNSs with 22 micronutrients/d (LNS) during pregnancy and a placebo (200 mg Ca/d), MMNs, or SQ-LNSs, respectively, for 6 mo postpartum. Weight, midupper arm circumference (MUAC), and triceps skinfold (TSF) thickness at 36 wk of gestation and 6 mo postpartum were analyzed, as were changes from estimated prepregnancy values. We assessed the adequacy of estimated gestational weight gain (GWG) by using Institute of Medicine (IOM) and International Fetal and Newborn Growth Standards for the 21st Century (INTERGROWTH-21st) guidelines.

**Results:** The estimated prepregnancy prevalence of overweight or obesity was 38.5%. By 36 wk of gestation, women (*n* = 1015) had a mean ± SD weight gain of 7.4 ± 3.7 kg and changes of −1.0 ± 1.7 cm in MUAC and −2.8 ± 4.1 mm in TSF thickness. The LNS group had a lower prevalence of inadequate GWG on the basis of IOM guidelines (57.4%) than the MMN (67.2%) but not the IFA (63.1%) groups (*P* = 0.030), whereas the prevalence of adequate (26.9% overall) and excessive (10.4% overall) GWG did not differ by group. The percentages of normal-weight women (in kg/m^2^: 18.5 < body mass index < 25.0; *n* = 754) whose GWG was less than the third centile of the INTERGROWTH-21st standards were 23.0%, 28.7%, and 28.5% for the LNS, MMN, and IFA groups, respectively (*P* = 0.36). At 6 mo postpartum, the prevalence of overweight or obesity was 45.3%, and the risk of becoming overweight or obese did not differ by group.

**Conclusion:** SQ-LNS supplementation is one potential strategy to address the high prevalence of inadequate GWG in women in settings similar to Ghana, without increasing the risk of excessive GWG. This trial was registered at clinicaltrials.gov as NCT00970866.

## Introduction

In many developing counties, pregnant and/or lactating women often suffer from vitamin and mineral deficiencies (1), commonly as a result of poor diet (2, 3), and the amounts of essential FAs available from the food supply may be below the minimum recommended levels for pregnant and lactating women (4). Vitamin and mineral deficiencies during pregnancy contribute to intrauterine growth retardation and pregnancy-related and delivery complications (5); therefore, efforts to combat these deficiencies are a global priority (6).

Iron and folic acid (IFA)^9^ supplementation (7) has been the main nutritional supplementation regimen for pregnant women in low-income countries for decades and has been found to reduce the risk of maternal anemia (8, 9) and iron deficiency (9) when compared with no iron or placebo. However, evidence from a recent Cochrane Review (10) suggests that multiple micronutrient (MMN) supplementation for pregnant women may be superior to iron supplementation with or without folic acid in terms of reducing the incidence of low birth weight, small-for-gestational-age, and stillbirth delivery.

Small-quantity lipid-based nutrient supplements (SQ-LNSs) providing 22 vitamins and minerals, including minerals not usually incorporated in MMN supplements, offer a relatively new strategy for delivering not only MMNs but also essential FAs to pregnant and lactating women. Published evidence from our study in Ghana (11) and another study from Bangladesh (12) showed a positive impact of prenatal SQ-LNS supplementation on fetal growth, particularly among women at a greater risk of delivering infants with intrauterine growth retardation. Given these results, interest in the use of maternal SQ-LNS supplementation in low-income communities might grow.

The Institute of Medicine (IOM) provides recommendations on gestational weight gain (GWG) based on prepregnancy BMI: underweight women have the greatest recommended weight gain range (12.5–18 kg), followed by normal-weight (11.5–16 kg) and then overweight (7–11.5 kg) and obese (5–9 kg) women (13). More recently, the International Fetal and Newborn Growth (INTERGROWTH-21st) Consortium for the 21st Century published GWG standards by using data from more diverse populations compared with the IOM recommendations (14). However, unlike the IOM recommendations, the INTERGROWTH-21st standards are appropriate for normal-weight women only, and therefore the former recommendations may have greater applicability.

Deviations from GWG recommendations have been associated with adverse consequences for both the mother and child: inadequate GWG is associated with an increased risk of low birth weight, whereas excessive GWG is associated with infant macrosomia and maternal postpartum weight retention and the development of overweight or obesity (13). Prolonged consumption of SQ-LNSs during pregnancy and lactation may affect women’s weight, but to our knowledge, so far only one study in Bangladesh (15) has examined this relation.

In this analysis, we examined several secondary outcomes of our International Lipid-based Nutrient Supplements (iLiNS)–DYAD efficacy trial in Ghana (11), a randomized controlled trial carried out by the iLiNS study group in which mother-child dyads were enrolled. We aimed to determine whether the consumption of SQ-LNSs was associated with differences in GWG or maternal anthropometric characteristics, including risk of overweight or obesity, when provided during pregnancy and the first 6 mo postpartum in a semi-urban setting in Ghana, where energy intake among women is generally not limiting and overweight and obesity are matters of growing concern.

## Methods

### 

#### Study setting, design, and participants.

We have previously described the setting, design, and participants of the iLiNS-DYAD efficacy trial in Ghana (11). Briefly, the study was conducted in a semi-urban settlement (Somanya-Odumase-Kpong area) in the Yilo Krobo and the Lower Manya Krobo districts ∼70 km north of Accra, Ghana. It was designed as a parallel, individually randomized, controlled trial with 3 equal-size groups. The study protocol was approved by the ethics committees of the University of California, Davis; the Ghana Health Service; and the University of Ghana Noguchi Memorial Institute for Medical Research and was registered at clinicaltrials.org (identifier NCT00970866).

Participants were pregnant women aged ≥18 y, at ≤20 wk of gestation, who were attending usual antenatal clinics in the 4 main health facilities in the area between December 2009 and December 2011, and who had antenatal cards complete with history and examination. A woman was excluded if any of the following applied: not residing in the area, intention to move out of the area within the next 2 y, milk or peanut allergy, unwillingness to receive fieldworkers or take study supplement, participation in another trial, gestational age >20 wk before completion of enrollment, and antenatal card indicating HIV infection, asthma, epilepsy, tuberculosis, or malignant disease.

#### Intervention.

We previously described (11) that after baseline assessments, eligible women were randomly assigned to receive 1 of 3 treatments: *1*) 60 mg Fe + 400 μg folic acid/d during pregnancy and placebo (200 mg Ca/d) during the first 6 mo postpartum (IFA group), *2*) MMN capsules containing 18 vitamins and minerals (including 20 mg Fe)/d during pregnancy and the first 6 mo postpartum (MMN group), and *3*) 20 g/d of SQ-LNSs containing micronutrients similar to those in the MMN supplement plus calcium, phosphorus, potassium, and magnesium as well as energy (118 kcal/d) and macronutrients (e.g., protein and essential FAs) during pregnancy and the first 6 mo postpartum (SQ-LNS supplement or LNS group). The SQ-LNS (individual 20-g sachets) was supplied by Nutriset S.A.S. and the IFA and MMN (10 capsules/blister pack) by DSM South Africa. We previously published the nutrient contents of all supplements (11) and the rationale for the concentrations of the nutrients (16).

Apart from iron, the vitamin and mineral contents of the MMN and SQ-LNS supplements were either 1 or 2 times the RDA for pregnancy or, in a few cases, the maximum amount that could be included in the supplement given technical and organoleptic constraints (16). Group allocations were developed by the study statistician with the use of a computer-generated (SAS version 9.4) scheme in blocks of 9, and randomization was performed by the study nurse who offered 9 sealed, opaque envelopes at a time, one of which was picked by the participant to reveal the allocation. Allocation information was securely kept by one field supervisor and the study statistician only.

At enrollment, the study nurse gave each woman a 2-wk supply of the assigned supplement with instructions on how to consume it (IFA and MMN, with water after a meal, 1 capsule/d; SQ-LNSs, mixed with any prepared food, one 20-g sachet/d) and a standard nutrition message (“Do not forget to eat meat, fish, eggs, fruits, and vegetables whenever you can; you still need these foods even as you take the supplement we have given you”) designed to reflect Ghana Health Service’s nutritional advice for women during pregnancy (17) and local food availability. Thereafter, fieldworkers visited women in their homes biweekly, at which time they delivered fresh supplies of supplement and monitored supplement intakes and morbidity (11).

After the women gave birth, fieldworkers visited them and their infants each week, but the delivery of supplement and monitoring of intakes and morbidity were done biweekly as before until women exited the study at 6 mo postpartum. During follow-up, women were told not to consume >1 capsule (IFA and MMN groups) or sachet (LNS group)/d, even if they forgot to take the supplement the previous day or days. Women were told to take their assigned supplement with them if they wanted to travel out of the study area. Those who would not return before the next biweekly visit were given an extra supply for the period they intended to be away.

To maintain blinding, the IFA and MMN supplements were color-coded (3 different colors for IFA and 3 for MMN supplements) and were therefore known to the study team and participants only by the colors. It was not possible to blind fieldworkers and participants to the IFA and MMN supplements compared with the SQ-LNS supplement due to their apparent differences, but the anthropometrists who measured the women were blinded to the group assignments, and no one apart from the study statistician had knowledge of group assignment until all preliminary analyses had been completed.

#### Outcome measures and procedures.

The outcome measures evaluated at 36 wk of gestation (last laboratory visit before delivery) were as follows: total gestational weight (kilograms), midupper arm circumference (MUAC; centimeters), and triceps skinfold (TSF) thickness (millimeters) gain; GWG per week; percentage adequacy of GWG achieved; percentage of women whose GWG was inadequate (less than the lower cutoff of recommendations), adequate (within the recommended range), or excessive (more than the upper cutoff of recommendations) according to the IOM GWG recommendations (13); and the percentage of normal-weight (in kg/m^2^; 18.5 < BMI < 25.0) women whose estimated GWG was less than the third centile or >97th centile of the INTERGROWTH-21st standards (14). Outcomes evaluated at 6 mo postpartum (maternal endpoint of the study) were weight, MUAC, TSF thickness, and BMI; change in weight, MUAC, TSF thickness, and BMI from prepregnancy; and the percentage of women who became overweight or obese out of those who were not overweight or obese at prepregnancy.

We collected background demographic and socioeconomic information at enrollment by using a questionnaire and completed anthropometric and laboratory assessments at enrollment, 36 wk of gestation, and 6-mo postpartum. Weight (Seca 874; Seca), height (Seca 217; Seca), MUAC, and TSF thickness (Holtain calipers) were measured with the use of standard procedures. Blood hemoglobin concentration was measured by HemoCue (HemoCue AG), and malaria parasitemia was assessed by using a kit (Vision Biotech) (11). As described previously (11), gestational age was determined mostly by ultrasound biometry (Aloka SSD 500).

#### Sample size and data analysis.

The sample size for the iLiNS-DYAD Ghana study (11) was based on detecting a small-to-moderate (18) effect size (Cohen’s d) of 0.3 between any 2 groups for any continuous outcome measure, with a 2-sided 5% test and 80% power. As previously reported (11), a total of 1320 pregnant women were enrolled into the study. We also previously reported (11) a temporary mislabeling of IFA and MMN capsules, as a result of which 170 women who had been assigned to the IFA group inadvertently received the MMN capsule either throughout pregnancy (*n* = 85) or during part of their pregnancy (*n* = 85) before receiving the intended IFA capsule for the rest of follow-up, and another 170 women assigned to the MMN group also received the IFA capsule either throughout pregnancy (*n* = 78) or during part of their pregnancy (*n* = 92) before receiving the intended MMN capsule. In this current analysis that covered both pre- and postnatal periods of the intervention, we included all of the women enrolled into the study without discarding any data from those who received the unintended supplements, because the unintended exposure occurred only in the prenatal period. We used this same approach in our previous publication (19) in which we evaluated the impact of the intervention spanning pre- and postnatal periods on the attained growth of 18-mo-old children. We estimated that the actual percentage of follow-up days (13%) during which the women in the IFA and MMN groups had the unintended exposure was relatively small (19), and in addition, no women in the LNS group were exposed to any other supplement apart from the intended SQ-LNSs. At 36 wk of gestation and 6-mo postpartum, we had anthropometric data for 1015 and 1073 women, respectively. With these sample sizes, we had >97% power to detect an effect size of 0.3 between any 2 groups for any continuous outcome at each of the time points.

We developed our statistical analysis plan and posted it on our website (www.ilins.org) before data analysis. The secondary outcomes in the present analysis were prespecified in the statistical analysis plan. Statistical analysis was performed on an intention-to-treat basis by using SAS for Windows Release 9.4. Thus, women were included in the analysis regardless of adherence to treatment. To address the protocol violation as a result of the consumption of mislabeled capsules by some women during pregnancy, we analyzed the data by using 2 scenarios as done previously (19). In the first, intervention groups were based on the supplement that women were intended to receive when they were enrolled, and in the second, intervention groups were based on the supplement women that actually received when they were enrolled.

The variables used to assess the outcome measures were obtained as follows: first, because it was not possible to obtain women’s prepregnancy weight (needed to apply the IOM GWG recommendations), we used a third-degree polynomial regression model with 1 predictor variable (gestational age at enrollment) to estimate prepregnancy weight on the basis of weight and gestational age at the time of enrollment. In this case, the shape of the true nonlinear response function of maternal weight to gestational age was unknown (or complex), and a polynomial function was a good approximation of the true function (20). The procedure was accomplished by first determining the best transformation of the weight at enrollment that achieved a normal distribution and regressing the transformed weight on gestational age, gestational age squared, and gestational age cubed in order to generate predicted and residual values. We then inspected the regression curve to determine the earliest gestational age before the CI expanded, assuming that weight gain before that time was minimal. Next, we determined the mean of the predicted values at the selected time point in early gestation, added this mean value to the residual for each individual, and then back-transformed the result to obtain the estimated prepregnancy weight for the individual. We used the same approach to estimate the prepregnancy MUAC and TSF thickness on the basis of the values measured at enrollment. We estimated prepregnancy BMI as estimated prepregnancy weight divided by the square of height measured at the time of enrollment.

We calculated the total GWG, gestational MUAC gain, and gestational TSF thickness gain by subtracting the estimated prepregnancy values from those measured at the last prenatal visit to the laboratory at ∼36 wk of gestation, an approach used by other investigators (21). The rate of GWG by 36 gestational weeks was calculated as total GWG divided by completed weeks of gestation (22). The percentage adequacy of GWG as a continuous variable was calculated by dividing the total GWG by the expected GWG (i.e., the amount of weight a woman was supposed to gain according to the IOM recommendation when her weight was measured at ∼36 wk of gestation) and multiplying the result by 100 (21). For the expected GWG, we used the following formula: expected GWG = expected first-trimester total weight gain + [(gestational age at the last weight measurement at ∼36 wk of gestation − 13 wk) × recommended rate of GWG for the second and third trimesters] (21, 23, 24). The expected first-trimester total weight gain was assumed to be 2 kg for underweight and normal-weight women, 1 kg for overweight women, and 0.5 kg for obese women (21); and the recommended rates of GWG for the second and third trimesters were 0.5, 0.4, 0.3, and 0.23 kg/wk for underweight, normal-weight, overweight, and obese women, respectively (25).

Because the IOM recommends a range of total GWG for each prepregnancy BMI group, we classified the percentage of weight-gain recommendations met as inadequate, adequate, or excessive (26). For each BMI-specific range, we divided the lower and upper limits of the recommended weight-gain range by the expected weight gain by 40 wk of gestation and multiplied the result by 100 to obtain the corresponding range of the recommended percentage of expected weight gain (26). For example, for women with a normal prepregnancy BMI, the expected GWG by 40 wk of gestation is as follows: 2.0 kg + [(40 wk − 13 wk) × 0.4 kg/wk] = 12.8 kg. For the IOM’s recommended total weight-gain range of 11.5–16 kg, the lower and upper limits of the corresponding range of the recommended percentage of expected weight gain are as follows: 11.5 kg/12.8 kg × 100 and 16 kg/12.8 kg × 100 = 90% − 125% of the 12.8-kg expected weight gain. Hence, we classified inadequate, adequate, and excessive weight gain as <90%, 90–125%, and >125% of recommendations, respectively. For a normal-weight woman who gained a total of 9.0 kg and whose weight was last measured at 37 wk of gestation, the expected GWG would be 11.7 kg and the percentage of recommendations met would be 9.0 kg/11.7 kg × 100 = 77%, which would be classified as inadequate GWG. Changes in weight, MUAC, TSF thickness, and BMI from prepregnancy to 6 mo postpartum were calculated by subtracting the estimated prepregnancy values, from the values measured at 6 mo postpartum.

To examine how the GWG assessment with the use of the IOM’s recommendation would compare with a similar assessment with the use of the INTERGROWTH-21st standards, we calculated the 3rd and the 97th centiles of expected GWG at the last antenatal measurement at ∼36 wk of gestation. For the third centile of GWG, we used the following formula:


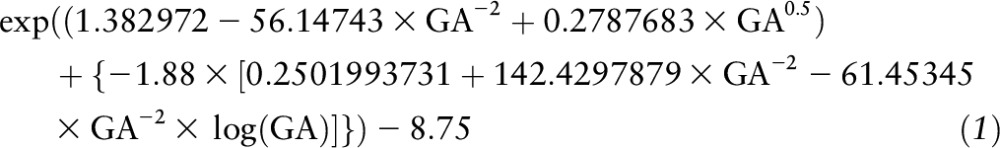


where GA = weeks of gestation at the last antenatal measurement (14). For the 97th centile of GWG, we replaced the −1.88 in the formula for the 3rd centile of expected GWG by 1.88 (14). We used actual GWG less than the calculated 3rd centile of the expected GWG as a proxy for inadequate GWG and actual GWG greater than the calculated 97th centile of expected GWG as a proxy for excessive GWG. Because the INTERGROWTH-21st standards are appropriate for normal-weight women, only women in our sample who had an estimated prepregnancy BMI between 18.5 and 24.99 were included in the analysis involving those standards.

We summarized the background characteristics at enrollment (11) and the number of days from the last prenatal measurements (∼36 wk of gestation) to delivery as means ± SDs or frequencies (percentage) by using the group assignment based on supplements that women were intended to receive when they were enrolled. At 36 wk of gestation and 6 mo postpartum, we calculated descriptive statistics for the overall sample, before comparing the 3 treatment groups by using general linear models (continuous outcomes) and logistic regression models (binary) with Tukey-Kramer adjustment for multiple comparisons. Along with the group comparisons, we calculated pairwise mean differences (continuous outcomes) and RRs (binary outcomes) with their 95% CIs and *P* values. RRs were calculated by using Poisson regression (27). These comparisons were performed twice, first without any covariate adjustments and then with adjustment for covariates that were significantly associated (*P* < 0.10) with the outcome in question in a bivariate analysis. Potential covariates were specified before analysis and included primiparity (yes or no), season at enrollment (wet or not wet), anemia (yes or no), age, gestational age at enrollment, and assets index, housing index, and Household Food Insecurity Access Scale score derived by using principal components analysis (28). Finally, as done previously (19) as a possible option for addressing the protocol violation, we performed a secondary analysis of the outcome variables, in which we combined women in the IFA and MMN groups to conduct a 2-group comparison with those who consumed the SQ-LNSs. We considered that for these assessments in a clinical trial, in which all of the outcomes were secondary, prespecified, and highly correlated, correcting for multiplicity was unnecessary (29).

Statistics in the texts are means ± SDs (continuous outcomes) or percentages (binary outcomes). Women’s adherence to supplement intake, defined as the percentage of follow-up days that women self-reported consuming the supplements (pregnancy/lactation) was as follows: 88.1%/85.7% for the IFA group, 87.0%/85.0% for the MMN group, and 83.7%/80.0% for the LNS group, as reported previously (30). Data on morbidity have yet to be analyzed, but we reported previously (11, 19) that serious adverse events were evenly distributed across the 3 groups.

## Results

### 

#### Overall sample.

The characteristics of the women enrolled, by intervention group, are shown in **Table 1**. On average, the women were ∼27 y of age, had 8 y of education, were generally not food insecure, and had a relatively low estimated rate of underweight (3.1%) and a high estimated rate of overweight and obesity (38.5%) at prepregnancy. These characteristics were generally balanced across the 3 groups. The number of days from the last prenatal measurement to delivery was 21.8 ± 10.5.

**TABLE 1:** Characteristics of women who participated in a randomized trial of IFA (pregnancy only), MMN (pregnancy and lactation), and LNS (pregnancy, lactation, and infancy) supplementation in a semi-urban setting in Ghana, by intervention group based on intended supplement at enrollment^1^

	Group		
Characteristics	IFA (*n* = 441)	MMN (*n* = 439)	LNS (*n* = 440)
Age, y	26.4 ± 5.6 (441)	26.9 ± 5.4 (439)	26.9 ± 5.6 (440)
Years of formal education	7.8 ± 3.5 (441)	7.6 ± 3.6 (439)	7.6 ± 3.9 (440)
Gestational age at enrollment, wk	16.0 ± 3.3 (440)	16.2 ± 3.2 (436)	16.1 ± 3.3 (435)
Asset index^2^	0.04 ± 1.03 (433)	0.06 ± 0.97 (431)	−0.09 ± 1.00 (432)
Housing index^2^	0.01 ± 1.00 (433)	0.01 ± 1.01 (431)	−0.01 ± 1.00 (432)
HFIAS score^3^	2.6 ± 4.4 (434)	2.7 ± 4.3 (431)	2.6 ± 4.0 (432)
Married or cohabiting, *n*/total *n* (%)	408/441 (92.5)	411/439 (93.6)	405/440 (92.0)
Primiparous women, *n*/total *n* (%)	156/441 (35.4)	143/439 (32.6)	147/440 (33.4)
Positive malarial RDT, *n*/total *n* (%)	37/440 (8.4)	42/439 (9.6)	54/440 (12.3)
Hemoglobin <100 g/L, *n*/total *n* (%)	57/440 (13.0)	68/439 (15.5)	60/440 (13.6)
Weight,^4^ kg	61.5 ± 11.6 (432)	61.5 ± 12.0 (429)	62.7 ± 12.3 (430)
BMI,^5^ kg/m^2^	24.4 ± 4.4 (432)	24.4 ± 4.3 (429)	24.8 ± 4.5 (430)
Underweight (<18.5), *n*/total *n* (%)	21/432 (4.9)	12/429 (2.8)	7/430 (1.6)
Overweight (25.0–29.9), *n*/total *n* (%)	113/432 (26.2)	112/429 (26.1)	129/430 (30.0)
Obese (≥30.0) *n*/total *n* (%)	48/432 (11.1)	46/429 (10.7)	49/430 (11.4)
MUAC,^4^ cm	28.4 ± 4.3 (432)	28.5 ± 4.2 (430)	28.9 ± 4.6 (430)
Triceps skinfold thickness,^5^ mm	19.8 ± 7.8 (432)	20.1 ± 7.9 (429)	20.7 ± 7.9 (430)
Days from last prenatal measurement to delivery	21.1 ± 9.9 (336)	21.7 ± 11.1 (357)	22.5 ± 10.4 (336)

1 Values are means ± SDs (*n*) unless otherwise indicated; *n* = 1320. Unless otherwise indicated, these characteristics were measured at the time of enrollment. *n*/Total *n* indicates the number of participants whose response was “yes” for the variable in question/total number of participants analyzed for the variable in question. HFIAS, Household Food Insecurity Access Scale; IFA, iron and folic acid; LNS, small-quantity lipid-based nutrient supplement; MMN, multiple micronutrient; MUAC, midupper arm circumference; RDT, Rapid Diagnostic Test (Clearview Malarial Combo; Vision Biotech; which detected *Plasmodium* *falciparum* and non–*P. falciparum* histidine-rich protein 2).

2 Proxy indexes for household socioeconomic status; higher values represent higher socioeconomic status.

3 HFIAS is a proxy indicator for household food insecurity (28); higher values represent higher food insecurity.

4 Prepregnancy values estimated from those measured at enrollment by using third-order polynomial regression, with gestational age at enrollment as the predictor variable.

5 Based on prepregnancy weight estimated from weight at enrollment by using polynomial regression, with gestational age at enrollment as the predictor variable.

On the basis of the estimated prepregnancy values, the overall GWG at 36 wk of gestation was 7.4 ± 3.7 kg, which was equivalent to 0.2 ± 0.1 kg/wk and 76.8% ± 43.0% of the expected weight gain according to the IOM’s weight-gain recommendation. Women lost 1.0 ± 1.7 cm in MUAC and 2.8 ± 4.1 mm in TSF thickness. The percentages of women in the sample who had inadequate, adequate, and excessive GWG at 36 wk of gestation by the IOM recommendations were 62.7%, 26.9%, and 10.4%, respectively. On average, 26.8% of women with an estimated normal BMI at prepregnancy had a total GWG of less than the third centile of the expected GWG on the basis of the INTERGROWTH-21st standards, but none had total GWG above the 97th centile of the expected GWG on the basis of the INTERGROWTH-21st standards.

By 6 mo postpartum, the overall mean ± SD (median) changes in women’s anthropometric indexes from the estimated prepregnancy values were as follows: +1.6 ± 4.9 (1.5) kg for weight, +0.4 ± 2.0 (0.3) cm for MUAC, +0.6 ± 5.0 (0.3) mm for TSF thickness, and +0.6 ± 1.9 (0.5) for BMI. The overall prevalence of underweight (BMI <18.5) remained relatively low (3.7%), whereas 45.3% of the women were overweight or obese.

#### Group comparisons.

The unadjusted results for the continuous outcome measures for the analysis based on groups according to the supplements women were intended to receive at enrollment are presented in **Table 2**. At 36 wk of gestation and 6 mo postpartum, the 3 groups did not differ in the unadjusted mean values of the continuous outcome measures, except for a tendency toward a greater percentage of adequacy of GWG in the LNS group than in the other groups (overall *P* = 0.09). In adjusted analysis controlling for covariates significantly associated with the outcome in question (details not shown), there were trends toward greater mean total estimated GWG (overall *P* = 0.07), GWG per week (overall *P* = 0.07), and percentage of adequacy of GWG (*P* = 0.08) for women in the LNS group compared with those in the other groups. Similar results for both unadjusted (**Supplemental Table 1**) and adjusted (data not shown) analyses were generally observed when groups were examined according to the supplements women actually received when they were enrolled.

**TABLE 2:** Unadjusted continuous anthropometric outcomes of women who participated in a randomized trial of IFA (pregnancy only), MMN (pregnancy and lactation), and LNS (pregnancy and lactation) supplementation in a semi-urban setting in Ghana, by intervention group based on intended supplement at enrollment^1^

					Comparison of					
	Group^2^				MMNs and IFA		LNSs and IFA		LNSs and MMNs	
Outcome variable	IFA (*n* = 441)	MMN (*n* = 439)	LNS (*n* = 440)	*P*^3^	Difference (95% CI)	*P*	Difference (95% CI)	*P*	Difference (95% CI)	*P*
36 wk of gestation^4^										
Total GWG, kg	7.3 ± 3.9 (331)	7.2 ± 3.6 (351)	7.7 ± 3.7 (331)	0.18	−0.2 (−0.9, 0.5)	0.79	0.3 (−0.3, 1.0)	0.48	0.5 (−0.1, 1.2)	0.16
Rate of GWG, kg/wk	0.2 ± 0.11 (331)	0.2 ± 0.10 (351)	0.2 ± 0.10 (331)	0.19	−0.0 (−0.0, 0.0)	0.76	0.0 (−0.0, 0.0)	0.51	0.0 (−0.0, 0.0)	0.16
Percentage of adequacy of GWG	76.1 ± 43.6 (331)	73.7 ± 41.0 (351)	80.8 ± 44.2 (331)	0.09	−2.4 (−10, 5.4)	0.75	4.7 (−3.1, 13)	0.33	7.1 (−0.6, 15)	0.08
Total MUAC change, cm	−1.1 ± 1.8 (331)	−1.1 ± 1.6 (352)	−0.9 ± 1.6 (332)	0.40	−0.0 (−0.3, 0.3)	0.98	0.1 (−0.2, 0.4)	0.54	0.2 (−0.1, 0.5)	0.41
Total TSF thickness change, mm	−2.7 ± 4.2 (331)	−2.9 ± 3.9 (351)	−2.7 ± 4.1 (332)	0.76	−0.2 (−1.0, 0.5)	0.76	−0.0 (−0.8, 0.7)	0.99	0.2 (−0.6, 0.9)	0.84
6 mo postpartum										
Weight, kg	63.6 ± 12.7 (355)	63.0 ± 13.1 (362)	64.2 ± 13.2 (356)	0.52	−0.6 (−2.9, 1.7)	0.81	0.5 (−1.8, 2.8)	0.85	1.1 (−1.2, 3.4)	0.48
MUAC, cm	29.1 ± 4.2 (355)	29.0 ± 4.3 (362)	29.2 ± 4.6 (356)	0.84	−0.1 (−0.9, 0.6)	0.92	0.1 (−0.7, 0.8)	0.98	0.2 (−0.6, 1.0)	0.83
TSF thickness, mm	20.9 ± 7. 6 (355)	20.8 ± 7.8 (362)	21.0 ± 7.8 (356)	0.95	−0.1 (−1.5, 1.3)	0.98	0.1 (−1.3, 1.4)	0.99	0.2 (−1.2, 1.5)	0.95
BMI, kg/m^2^	25.2 ± 4.7 (355)	25.0 ± 4.7 (362)	25.4 ± 4.8 (356)	0.67	−0.2 (−1.0, 0.7)	0.90	0.2 (−0.7, 1.0)	0.89	0.3 (−0.5, 1.2)	0.64
Change from prepregnancy^5^										
Weight, kg	1.9 ± 4.7 (348)	1.40 ± 5.1 (356)	1.4 ± 4.8 (351)	0.33	−0.5 (−1.4, 0.4)	0.39	−0.5 (−1.3, 0.4)	0.40	0.0 (−0.9, 0.9)	1.00
MUAC, cm	0.6 ± 2.0 (348)	0.3 ± 2.0 (357)	0.3 ± 2.1 (351)	0.12	−0.2 (−0.6, 0.1)	0.24	−0.3 (−0.6, 0.1)	0.14	−0.0 (−0.4, 0.3)	0.95
TSF thickness, mm	0.9 ± 4.9 (348)	0.5 ± 5.0 (356)	0.4 ± 5.0 (351)	0.30	−0.5 (−1.3, 0.4)	0.43	−0.5 (−1.4, 0.3)	0.33	−0.1 (−1.0, 0.8)	0.98
BMI, kg/m^2^	0.7 ± 1.9 (348)	0.6 ± 2.0 (356)	0.5 ± 1.9 (351)	0.36	−0.2 (−0.5, 0.2)	0.45	−0.2 (−0.5, 0.2)	0.41	−0.0 (−0.4, 0.3)	1.00

1 *n* = 1320. IFA group: women were assigned to receive 60 mg Fe + 400 mg folic acid/d during pregnancy and placebo (200 mg Ca/d) during the first 6 mo postpartum; MMN group: women were assigned to receive 18 vitamins and minerals (including 20 mg Fe)/d during pregnancy and the first 6 mo postpartum; LNS group: women were assigned to receive 20 g SQ-LNS/d with the same micronutrients as the MMN group + calcium, phosphorus, potassium, and magnesium as well as macronutrients during pregnancy and the first 6 mo postpartum. Results are based on ANOVA (SAS PROC GLM). GWG, gestational weight gain; IFA, iron and folic acid; LNS, lipid-based nutrient supplement; MMN, multiple micronutrient; MUAC, midupper arm circumference; SQ-LNS, small-quantity lipid-based nutrient supplement; TSF, triceps skinfold.

2 Values are means ± SDs (*n*). Except for weight, MUAC, TSF thickness, and BMI at 6 mo postpartum, mean ± SD values are based on prepregnancy values estimated from those measured at enrollment by using third-order polynomial regression with gestational age at enrollment as the predictor variable.

3 *P* values for comparison of means ± SDs between the 3 groups, with Tukey-Kramer adjustment for pairwise comparisons.

4 Observed total GWG and MUAC and TSF thickness changes were calculated by subtracting the estimated prepregnancy weight, MUAC, and TSF thickness, respectively, from the weight, MUAC, and TSF thickness measured at the last prenatal visit (21). Estimated prepregnancy weight, MUAC, and TSF thickness were calculated from baseline values by using third-order polynomial regression with gestational age at enrollment as the predictor variable. Rate of GWG was calculated as total GWG at the last prenatal measurement divided by completed weeks of gestation. Percentage of adequacy of GWG [continuous: percentage of weight-gain recommendations met (26)] was calculated by dividing the observed total GWG by the expected GWG according to the Institute of Medicine’s recommended ranges (25) up to the woman’s last prenatal measurement. Expected GWG = expected first-trimester total weight gain + [(gestational age at the time of last weight measurement − 13 wk) × recommended rate of GWG for the second and third trimesters] (21, 23, 24).

5 Change from prepregnancy to 6 mo postpartum was calculated by subtracting the estimated prepregnancy values from the values measured at 6 mo postpartum.

When comparing women in the IFA and MMN groups combined with those in the LNS group (**Supplemental Table 2**), the trends toward a greater mean total GWG (*P* = 0.08) and GWG per week (*P* = 0.09) for the LNS group paralleled those observed in the 3-group comparison and the mean ± SD percentage of adequacy of GWG in the LNS group was significantly greater (*P* = 0.038) than that for the combined IFA + MMN group.

The unadjusted results for the binary outcome measures for the analysis that examined groups according to the supplements women were intended to receive at enrollment are presented in **Table 3**. At 36 wk of gestation, the 3 groups did not differ significantly in the percentage of women with adequate or excessive GWG according to the IOM’s recommendations, but the percentage of women who had inadequate GWG was significantly lower in the LNS group (57.4%) than in the MMN (but not the IFA) group [67.2%; overall *P* = 0.030 corresponding to RR = 0.85 (95% CI: 0.74, 0.98; *P* = 0.023)]. Among women with an estimated normal BMI at prepregnancy, the point estimate of the percentage of those whose total GWG was less than the third centile of the expected GWG based on the INTERGROWTH-21st standards was 23% for the LNS group compared with 28.5% for the IFA group and 28.7% for the MMN group, although the differences were not significant. The percentage of women with a normal BMI at prepregnancy who became overweight or obese by 6 mo postpartum also did not differ significantly between the 3 groups. These results remained unchanged after controlling for prespecified covariates significantly associated with the outcomes (details not shown). As observed for the continuous outcome variables, similar results for the binary outcome variables were found in the analysis based on the supplements women actually received when they were enrolled (unadjusted, **Supplemental Table 3**; adjusted, data not shown).

**TABLE 3:** Unadjusted binary anthropometric outcomes of women who participated in a randomized trial of IFA (pregnancy only), MMN (pregnancy and lactation), and LNS (pregnancy and lactation) supplementation in a semi-urban setting in Ghana, by intervention group based on intended supplement at enrollment^1^

					Comparison of^4^					
	Group^2^				MMNs and IFA		LNSs and IFA		LNSs and MMNs	
	IFA (*n* = 441)	MMN (*n* = 439)	LNS (*n* = 440)	*P*^3^	RR (95% CI)	*P*	RR (95% CI)	*P*	RR (95% CI)	*P*
GWG, %										
Inadequate^5^	63.1 (57.8, 68.2) [331]	67.2 (62.1, 72.0) [351]	57.4 (52.0, 62.6) [331]	0.030	1.1 (0.9, 1.2)	0.50	0.9 (0.8, 1.1)	0.29	0.9 (0.7, 1.0)	0.023
Adequate^6^	25.7 (21.3, 30.7) [331]	24.2 (20.0, 29.0) [351]	31.1 (26.4, 36.3) [331]	0.11	0.9 (0.7, 1.3)	0.90	1.2 (0.9, 1.6)	0.27	1.3 (1.0, 1.7)	0.11
Excessive^7^	11.2 (8.2, 15.1) [331]	8.5 (6.0, 12.0) [351]	11.5 (8.5, 15.4) [331]	0.38	0.8 (0.4, 1.3)	0.48	1.0 (0.6, 1.7)	0.99	1.3 (0.8, 2.3)	0.41
<10th centile of expected GWG^8^	28.5 (22.5, 35.4) [186]	28.7 (23.0, 35.2) [209]	23.0 (17.5, 29.6) [187]	0.36	1.0 (0.7, 1.5)	1.00	0.8 (0.5, 1.2)	0.45	0.8 (0.5, 1.2)	0.40
>97th centile of expected GWG^8^	0	0	0	—	—	—	—	—	—	—
Developed overweight or obesity by 6 mo postpartum^9^	20.9 (15.8, 27.2) [196]	15.7 (11.5, 21.2) [216]	17.2 (12.6, 23.0) [204]	0.37	0.8 (0.5, 1.2)	0.36	0.8 (0.5, 1.3)	0.60	1.1 (0.6, 1.8)	0.92
Developed obesity by 6 mo postpartum^10^	0.5 (0.1, 3.5) [196]	0.0 (0.0, 100) [216]	0.0 (0.0, 100) [204]	1.00	0.0 (0.0, 0.0)	1.00	0.0 (0.0, 0.0)	1.00	1.0 (0.8, 1.3)	1.00

1 *n* = 1320. IFA group: women were assigned to receive 60 mg Fe + 400 mg folic acid/d during pregnancy and placebo (200 mg Ca/d) during the first 6 mo postpartum; MMN group: women were assigned to receive 18 vitamins and minerals (including 20 mg Fe)/d during pregnancy and the first 6 mo postpartum; LNS group: women were assigned to receive 20 g SQ-LNS/d with the same micronutrients as the MMN group + calcium, phosphorus, potassium, and magnesium as well as macronutrients during pregnancy and the first 6 mo postpartum. Results are based on logistic regression (SAS PROC GLIMMIX). GA, gestational age; GWG, gestational weight gain; IFA, iron and folic acid; IOM, Institute of Medicine; LNS, lipid-based nutrient supplement; MMN, multiple micronutrient; SQ-LNS, small-quantity lipid-based nutrient supplement.

2 Values are percentages (95% CIs) [*n*]. Percentages (95% CIs) are based on prepregnancy values estimated from those measured at enrollment by using third-order polynomial regression with GA at enrollment as the predictor variable.

3 *P* values for comparisons between all 3 groups, with Tukey-Kramer adjustment for pairwise comparisons.

4 RRs (95% CIs) and their *P* values are based on Poisson regression (27).

5 Below the lower cutoff of the IOM’s recommended range (13).

6 Within the IOM’s recommended range (13).

7 Above the upper cutoff of the IOM’s recommended range (13).

8 Based on INTERGROWTH-21st standards for women whose estimated prepregnancy weight was normal (BMI: 18.50–24.99). The third centile of expected GWG was calculated as follows: exp((1.382972 − 56.14743 × GA^−2^ + 0.2787683 × GA^0.5^) + {−1.88 × [0.2501993731 + 142.4297879 × GA^−2^ − 61.45345 × GA^−2^ × log(GA)]}) − 8.75, where GA = weeks of gestation at the last antenatal measurement (14). The 97th centile of expected GWG was calculated by replacing the −1.88 in the formula for the third centile of expected GWG by 1.88 (14). No woman in the entire sample had total GWG above the 97th centile of the expected GWG based on the INTERGROWTH-21st standards.

9 Proportion of women with normal prepregnancy BMI who became overweight or obese by 6 mo postpartum.

10 Proportion of women with normal prepregnancy BMI prepregnancy who became obese by 6 mo postpartum.

In the 2-group comparison (**Supplemental Table 4**), the percentage of women with inadequate GWG was significantly lower (*P* = 0.016) and the percentage of women with adequate GWG was significantly greater (*P* = 0.038) in the LNS group than in the IFA and MMN groups combined. In addition, the results from the analysis that used the INTERGROWTH-21st standards mimicked those for the 3-group comparison.

## Discussion

In the semi-urban setting in Ghana where the iLiNS-DYAD study was conducted, we found that at 36 wk of gestation, women in the LNS, MMN, and IFA groups did not differ significantly in most of the secondary anthropometric (weight, MUAC, and TSF thickness) outcomes measured. However, by IOM recommendations, the prevalence of inadequate GWG based on estimated prepregnancy values was significantly lower in the LNS group than in the MMN group; and in a 2-group analysis, the mean ± SD percentage of adequacy of GWG was significantly greater, the prevalence of inadequate GWG was significantly lower, and the prevalence of adequate GWG was significantly greater in the LNS group than in the non-LNS group (IFA and MMN groups combined). At 6 mo postpartum, the groups did not differ in weight, MUAC, TSF thickness, BMI, or changes in these indexes from estimated prepregnancy values, in the percentage of women with excessive GWG, or the percentage of women who had a normal BMI at prepregnancy but who became overweight or obese by 6 mo postpartum.

An estimate of each woman’s prepregnancy BMI was a prerequisite for applying the IOM’s GWG recommendations. Previously, the proxies used for prepregnancy weight or BMI have included weights measured at various times including the following: first antenatal booking (31), within the previous 12 mo of pregnancy (32), in the first trimester (33, 34), and during pregnancy regardless of gestational age (35). As previously noted (36), each of these has its own limitations. We considered our approach to estimate prepregnancy weight or BMI (and MUAC and TSF thickness) with the use of polynomial regression (20, 37, 38) to be a good option, for 2 reasons: first, women in this population usually do not know their prepregnancy weight. Second, at a mean gestational age of 16.1 wk when women were enrolled, the estimated prepregnancy weight of 61.9 ± 11.9 kg implied that the women, on average, had potentially gained 0.7 kg by the time of enrollment and we decided not to ignore this change, as well as those in MUAC and TSF thickness that had occurred. The IOM assumes that women gain 0.5–2.0 kg during the first trimester (13), and therefore the average estimated 0.7-kg gain by the time of enrollment would be considered reasonably unbiased.

The estimated prepregnancy BMI for women in our study, who were aged 18–45 y, was similar to the mean BMI (24.8) reported for women aged 15–49 y (excluding those who were pregnant or had given birth the previous 2 mo) in the 2014 Ghana Demographic and Health Survey (39) for the region of Ghana (Eastern Region) where our study was conducted. Thus, we believe the estimation of prepregnancy weight, MUAC, and TSF thickness did not generally bias our results.

The losses in MUAC and TSF thickness in the sample at 36 wk of gestation compared with estimated prepregnancy values appear to be characteristic of pregnancy, during which fat is mobilized from the upper body and preferentially deposited over the hips, back, and upper thighs (13, 40). Our results showing the estimated prevalence of inadequate GWG according to IOM recommendations (63%) and the percentage of normal-weight women whose estimated GWG was less the third centile of expected GWG according to the INTERGROWTH-21st standards (27%) suggest that low GWG might be a problem in this setting. This observation is consistent with the area being located in the Eastern Region, which has the highest prevalence of low birth weight (14%) among the 10 regions of Ghana (39). Low GWG is associated with low birth weight (41). Elsewhere in Ghana, a low-GWG prevalence of 50% based on the IOM’s guidelines was reported. Thus, SQ-LNS supplementation during pregnancy might help, albeit modestly, to reduce low GWG in this setting.

Two previous reports may be relevant in relation to our results. In a large cluster-randomized trial in Bangladesh in which women received IFA or SQ-LNSs with nutrient contents similar to those provided in this study (15), the provision of SQ-LNSs increased maternal weight gain and MUAC only in certain subgroups (e..g, women ≥25 y of age) but, as in Ghana, this was not associated with excessive GWG. In a Cochrane Review (42), balanced protein-energy supplements (i.e., supplements in which protein provides <25% of the total energy content) given to pregnant women increased mean weight gain per week compared with no supplementation, but the daily amounts of energy in those supplements were substantially larger (>400 kcal) than the amount present in SQ-LNSs (118 kcal).

Our finding that SQ-LNS supplementation decreased the prevalence of inadequate GWG but was not associated with excessive GWG or risk of overweight and obesity at 6 mo postpartum implies a favorable response to the use of SQ-LNSs, particularly for populations such as that in our study setting where the nutrition transition is underway. The high prevalence of inadequate GWG observed in this cohort, coupled with the 12% prevalence of low birth weight that we previously reported (11), suggest that low energy intake during pregnancy may be an issue of concern in this population, even though a substantial percentage of the women were overweight before pregnancy. Low energy intake and a low intake of dairy products have been identified as important predictors of inadequate GWG (43–45), along with other biological or metabolic factors (27). SQ-LNSs provided a small amount of extra energy, and milk powder was one of the ingredients, which may have contributed to the reduced prevalence of inadequate GWG in the LNS group. The results reported herein are consistent with the previously reported greater mean birth weight and the lower prevalence of low birth weight of infants in the LNS group (11).

Because the consumption of SQ-LNSs was not associated with greater total GWG or a higher prevalence of excessive GWG in our sample, it is not surprising that it was also not associated with a greater risk of becoming overweight or obese by 6 mo postpartum. Women’s weight retention by 6 mo postpartum depends not only on dietary factors but also on physical activity and breastfeeding practices. In the study setting, where most women were relatively active (compared with those in, e.g., urban Accra) and nearly all women breastfed their infants during the first 6 mo postpartum (39), the extra energy provided by SQ-LNSs did not exacerbate the already high prevalence (estimated 38.5% at prepregnancy) of maternal overweight. This is reassuring, but we cannot be sure that this result is generalizable to other contexts.

The iLiNS-DYAD Ghana study has several strengths, including the use of a fully randomized design and having control groups. In addition, all anthropometrists were well trained and standardized (46) every 6 mo during data collection. Study weaknesses include the following: the inability to fully blind all study staff and participants to the supplementation allocation (due to the obvious differences between the IFA and MMN capsules compared with the SQ-LNS sachets); lack of data on women’s prepregnancy weight, which then had to be estimated; and the exposure of some women to both IFA and MMN supplements during part of pregnancy. It is possible that some of our findings may be due to chance because of multiple testing (29). However, all of the anthropometrists and data analysts were fully blinded to the group assignments until analyses were completed, and no women in the LNS group were exposed to any other supplement apart from the intended LNS. We therefore believe that the study weaknesses do not bias the finding of no association of SQ-LNS consumption with excessive GWG or increased risk of overweight or obesity by 6 mo postpartum. We conclude that daily SQ-LNS supplementation is one potential strategy to address the high prevalence of inadequate GWG in similar settings, without increasing the risk of excessive GWG or of becoming overweight or obese by 6 mo postpartum.
